# Drug Design by Pharmacophore and Virtual Screening Approach

**DOI:** 10.3390/ph15050646

**Published:** 2022-05-23

**Authors:** Deborah Giordano, Carmen Biancaniello, Maria Antonia Argenio, Angelo Facchiano

**Affiliations:** 1 National Research Council, Institute of Food Science, Via Roma 64, 83110 Avellino, Italy; deborah.giordano@isa.cnr.it (D.G.); maria.antonia.argenio@gmail.com (M.A.A.); 2 Doctorate School in Computational and Quantitative Biology, University of Naples “Federico II”, 80100 Naples, Italy; carmenbnl93@gmail.com

**Keywords:** structure-based pharmacophore modeling, ligand-based pharmacophore modeling, virtual screening, drug discovery, bioinformatics, computational biology

## Abstract

Computer-aided drug discovery techniques reduce the time and the costs needed to develop novel drugs. Their relevance becomes more and more evident with the needs due to health emergencies as well as to the diffusion of personalized medicine. Pharmacophore approaches represent one of the most interesting tools developed, by defining the molecular functional features needed for the binding of a molecule to a given receptor, and then directing the virtual screening of large collections of compounds for the selection of optimal candidates. Computational tools to create the pharmacophore model and to perform virtual screening are available and generated successful studies. This article describes the procedure of pharmacophore modelling followed by virtual screening, the most used software, possible limitations of the approach, and some applications reported in the literature.

## 1. Introduction

Computer-Aided Drug Discovery (CADD) investigates molecular properties to develop novel therapeutic solutions by way of computational tools and data resources. In its broadest meaning, it includes computational approaches for designing or selecting compounds as potential candidates before they are synthesized and tested for their biological activity [1]. Bioinformatics and computational tools offer an in silico approach to reducing costs and times, i.e., the factors that influence the progress of the research and, in the specific field of drug development, limit the possibilities of fighting more pathologies. To date, in vitro screening is expensive and time-consuming, and alternatives are highly desirable. Virtual Screening (VS) is a CADD method that involves in silico screening of a library of chemical compounds, to identify those that are most likely to bind to a specific target [2]. In this way, it is possible to reduce the impact of these limiting factors on drug discovery, meeting the needs due to health emergencies, as well as the spread of personalized medicine. This process can be speeded up using pharmacophore models used as the query with which compound libraries can be searched to pull out molecules of interest with desired properties. Indeed, pharmacophore-based methods are widely used tools in CADD and are of great interest in the chemo-informatics field [2], since they find many applications in drug discovery projects including not only the virtual screening but also scaffold hopping, lead optimization, ligand profiling, target identification, multi-target drug or de novo drug design.

The concept of a pharmacophore was coined in the 19th century when Langley first suggested that certain drug molecules might act on particular receptors. Only later, with the discovery of Salvarsan by Paul Elrich, the selectivity of drug–target interactions was recognized. Several years of experimentation confirmed its therapeutic effect. This discovery had found support in the statement of Emil Fisher who, following his research, had defined the concept "Lock & Key" in 1894, according to which a ligand and its receptor fits like a key with its lock to interact with the on top of each other through a chemical bond [3]. 

Hence, historically the term “pharmacophore” was used to indicate the functional or structural capacity of a compound with specific characteristics towards a biological target [4]. 

Schueler provided the basis for our modern understanding of a pharmacophore, defined by the International Union of Pure and Applied Chemistry (IUPAC) as "the ensemble of steric and electronic features that is necessary to ensure the optimal supra-molecular interactions with a specific biological target structure and to trigger (or to block) its biological response" [3,5,6].

**Figure 1:**
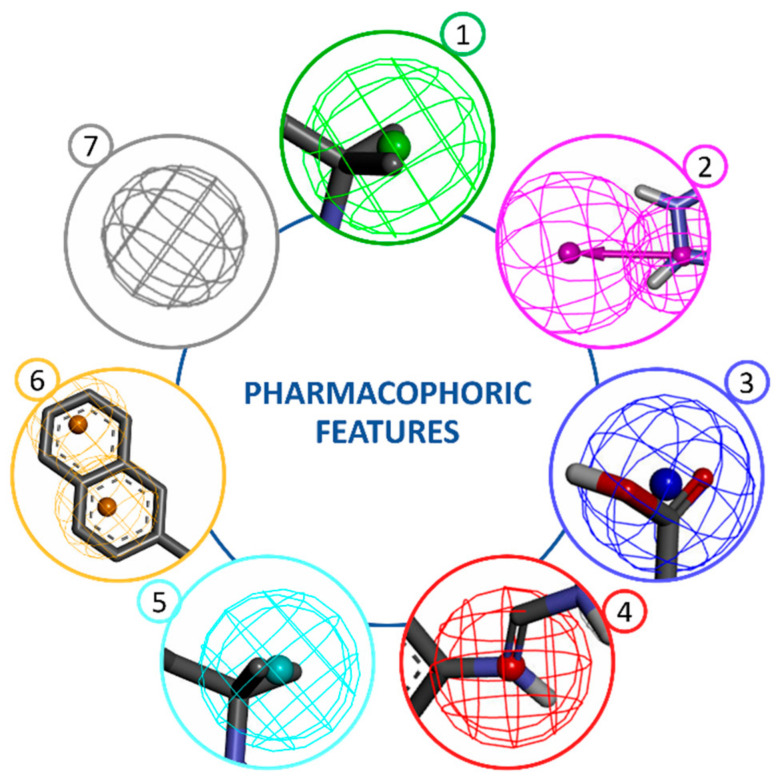
Pharmacophoric features. Main pharmacophoric feature types are represented by geometric entities and include: 1—hydrogen bond acceptor (HBA), 2—hydrogen bond donor (HBD), 3—negative ionizable (NI), 4—positive ionizable (PI), 5—hydrophobic (H), 6—aromatic (AR), 7—exclusion volume (XVOL).

Pharmacophoric modelling is based on the theory that having common chemical functionalities, and maintaining a similar spatial arrangement, leads to biological activity on the same target. The chemical characteristics of a molecule capable of creating interactions with its ligand are represented in the pharmacophoric model as geometric entities such as spheres, planes and vectors. The most important pharmacophoric feature types are: hydrogen bond acceptors (HBAs); hydrogen bond donors (HBDs); hydrophobic areas (H); positively and negatively ionizable groups (PI/NI); aromatic groups (AR); and metal coordinating areas (Figure 1). Additional size restrictions in the form of a shape or exclusion volumes (XVOL)—forbidden areas—can be added to represent the size and the shape of the binding pocket [7].

Since the models themselves do not focus on actual atoms, but on chemical functionalities, they are good tools in recognizing similarities between molecules. Pharmacophore activity is independent of the scaffold, and this explains why similar biological events can be triggered by chemically divergent molecules. The use of these models as a query allows performing searches in the large libraries of compounds made available on the computational platform in order to select molecules of interest for the next vs. or in the chemo-informatics field [7]. 

Pharmacophore models can be generated using two different approaches depending on the input data employed for model construction, which are, namely, “structure-based” and “ligand-based” pharmacophore modelling. The structure-based approach uses the structural information of the target proteins like enzymes or receptors, to identify compounds that can potentially be used as a drug. On the other hand, the ligand-based approach consists of the development of 3D pharmacophore models and modelling quantitative structure-activity relationship (QSAR) or quantitative structure-property relationship (QSPR), using only the physicochemical properties of known ligand molecules for drug development. The choice of the best approach to use depends on several factors such as data availability, data quality, computational resources and also the intended use of the generated pharmacophore models. In the following paragraphs, we describe both strategies and their implementation in virtual screening is provided below to guide the non-experts on this topic in their application by explaining the basic concepts these methods are based on.

## 2. Structure-Based Pharmacophore Modelling

**Figure 2:**
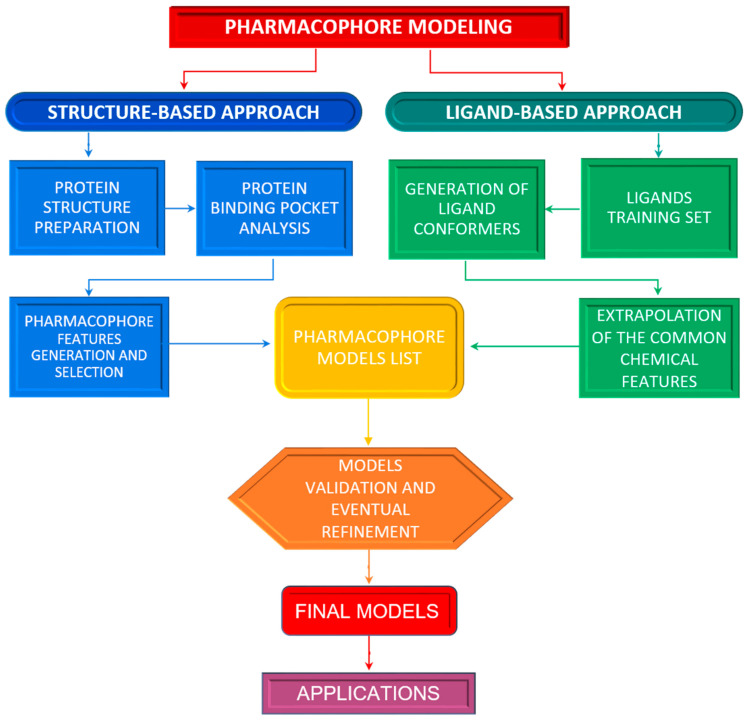
Pharmacophore modeling workflow. A pharmacophore model can be generated using two approaches. In the structure-based approach (on the left side), the structural information of the macromolecular target, alone or bounded to a ligand, is evaluated for its correctness and used to analyze the ligand binding sites in order to derive and select ligand complementary characteristics composing the pharmacophore models. In the ligand-based approach (on the right side), the first step is the construction of a training set made up of known active ligands structures. From each of them, conformers are generated and then used to extrapolate common features that are combined in different pharmacophore models. The resultant models undergo a validation and refinement procedure to obtain the final models exploitable for practical applications.

The structure-based approach owns its name to the fact that the three-dimensional structure of a macromolecule target is the essential prerequisite to obtaining a structure-based pharmacophore. The 3D structure of a protein provides significant details at the atomic level that can be very useful for the design or discovery of new drugs. As previously outlined, a pharmacophore is an abstract picture showing the stereo-electronic features, which make a ligand bioactive toward a specific target, and this type of information can be extracted from the 3D structure of the protein target in its holo or apo form. Typically, the workflow of the structure-based approach (Figure 2, left side of the flow) consists of different steps: protein preparation, identification or prediction of ligand binding site, pharmacophore features generation, and the selection of relevant features for ligand activity [8,9]. 

The 3D structure of the target or the ligand–target complex is the required starting point of this methodology. The RCSB Protein Data Bank (PDB) [www.rcsb.org] (accessed on 20 May 2022) includes thousands of protein structures at high resolution alone or in the presence of a bound ligand, mainly solved by X-ray crystallography or NMR spectroscopy. In case experimental structural data are lacking, computational techniques such as homology modelling [10] may be an alternative strategy to retrieve a 3D model and machine learning-based methods are successfully applied to retrieve protein structures, a powerful example is ALPHAFOLD2 [11]. Molecular docking is another method to study in silico the interaction of a known active compound towards a specific receptor and derive protein-ligand complexes from the most favorable binding poses [12].

A practical tip for those who want to use this approach is to perform a deep analysis of the quality of input data before using the target structure since it directly influences the quality of the pharmacophore model. For this reason, the protein structure preparation is the first step to carry out by evaluating, for instance, the residues’ protonation states, the position of hydrogen atoms (that are absent in X-ray solved structures), the presence of non-protein groups which may have or not some functional roles, eventual missing residues or atoms, the stereochemical and energetic parameters accounting for the general quality and biological-chemical sense of the investigated target, also in the case of experimentally solved structures that may contain errors [8]. 

Once the target structure is identified and critically evaluated, ligand-binding site detection is the next crucial step. This can be manually inferred by analyzing the area including residues suggested to have a key role from experimental data such as site-directed mutagenesis or X-ray structures of the protein co-crystallized with ligands, but it also requires time and expert knowledge of the target and ligands. Nowadays this purpose can be easily and quickly achieved using bioinformatics tools based on different methods which inspect the protein surface to search for potential ligand-binding sites according to various properties (evolutionary, geometric, energetic, statistical, or a combination of them), and which are particularly useful if the ligand information is not available [13]. Examples of computer programs developed for this purpose are GRID [14] and LUDI [15]. The first one, as suggested by the name, is a grid-based method that uses different molecules or functional groups to sample a specific protein region defined with a regular grid to identify grid points with which they make energetically favorable interactions, thus generating molecular interaction fields [14]. The second one predicts potential interaction sites using the knowledge from distributions of non-bonded contacts in experimental structures or geometric rules [15]. 

Since the goal of the structure-based method is to generate a pharmacophore model from the interactions between an active molecule (the ligand) and its protein target (enzyme or receptor), the characterization of the ligand-binding site can be used to derive a map of interaction and to build accordingly one or more pharmacophore hypotheses describing the type and the spatial arrangement of the chemical features required for a ligand to interact with the residues of the binding region. Initially, many features are detected with this approach and therefore only those that are essential for ligand bioactivity should be selected and incorporated into the final model to have a more reliable and selective pharmacophore hypothesis [9,16]. This operation can be accomplished in different ways, such as removing features that do not strongly contribute to the energy binding, identifying the most conserved interaction if multiple protein–ligands structures exist, preserving residue with key functions from sequence alignments or variation analysis, and incorporating spatial constraints from the receptor information [8].

If the structure of a protein–ligand complex is available, the pharmacophore features generation and selection can be achieved more accurately. The 3D information of the ligand in its bioactive conformation directs the identification and the spatial disposition of the pharmacophore features in correspondence with its functional groups directly involved in the interactions with the target. Moreover, the presence of the receptor allows accounting for spatial restrictions from the binding site shape through the addition of the exclusion volumes. In this ideal situation, pharmacophore models of high quality can be obtained. In case the available structural data do not include a bound ligand, the pharmacophore modelling depends only on the target structure which can be analyzed to detect all possible ligands interaction points in the binding sites and, therefore, to compute the complementary features that a ligand should match to potentially bind the receptor. However, in the absence of a ligand counterpart, several pharmacophoric features are calculated and approximately positioned resulting in less accurate models that should be manually refined [3,16,17].

## 3. Ligand-Based Pharmacophore Modelling

So far, we have mentioned the importance of the availability of the structure for modelling pharmacophores suitable to accommodate the best interaction features. However, even if this still represents one of the best strategies for modelling a pharmacophore, often the target molecule is not available. To overcome this problem, an alternative approach to structural-based modelling is represented by ligand-based pharmacophore modelling.

The starting point of this strategy is the knowledge of active compounds, which bind the same protein target with a similar orientation [4] that could allow the extraction of chemical features in common and, therefore, the pharmacophore construction. Due to the unknown bioactive conformations of the input compounds, a critical step, before the extraction of the shared features, is the generation of ligand conformers, so that, from each set of conformers, at list one should correspond to the bioactive conformation of the ligand [3].

In more detail, two datasets are necessary to generate a ligand-based pharmacophore, i.e., a training set and a test set. The training set composition differs according to the algorithm employed and the data availability, varying from the simplest, composed of at least two active compounds, to the most complex, for which it is possible to set multiple compounds with a different activity. The test set, instead, should contain as many active and inactive structurally different compounds as possible, preferring the inclusion of experimentally confirmed inactive compounds and compounds judged as active by experimentally proven direct interaction and with suitable activity cutoffs (i.e., low EC50/IC50 and high binding affinity values). In the case of unknown inactive ligands, decoy molecules could be used [6], which are available from a specific repository such as DUD (Directory of Useful Decoys) [18] or by generating them by service as DUD-E [19]. In addition to what could be extrapolated from pertinent literature, the source of molecules to be employed in the construction of the dataset could be chemical databases such as OpenPHACTS [20], ChEMBL [21] or Drugbank [22], for compounds with a target-based activity, ChEMBL, PubChem Bioassay [23] and Tox21 [24] for research on the inactive molecules. In principle, all the algorithms appointed to the ligand-based pharmacophore modelling, starting from the active compounds of the training set, extrapolate from them common chemical functionalities, related for instance to the presence of hydrogen bond donors or acceptors, of a positively or negatively charged ionizable group, of aromatic ring systems or hydrophobic areas, and use these features for model design. This step is followed by an improvement of the pharmacophore model including shape features and spatial constrains which take care of specific regions occupied by inactive molecules [25]. The pharmacophore models obtained are ranked by the best ability to fit the active molecules but not the inactive ones. The best models obtained from this first phase are chosen for the validation and refinement using the test set. The purpose of this second phase is to evaluate if the generated models can fit most of the active molecules rejecting the inactive ones, to select only models which show the major sensitivity and specificity. A summary of the described process is described in Figure 2 (right side of the flow).

Despite the accuracy of the pharmacophore construction being highly dependent on the quality of the training set, its final composition is conditioned by the algorithm used for modelling [7]. In general, ligand-based pharmacophore modelling could be performed following two main strategies: the common feature pharmacophore and the QSAR based pharmacophore.

In the common feature approach, generally, an algorithm of alignment is involved, its purpose is basically to extrapolate and align specific and common features: starting from a defined feature for each active element the algorithm combines it with the corresponding features of the other molecules, from this alignment a novel feature, combination of the previous, is generated; reiterating this procedure for each set of overlapping features a pharmacophore of shared feature is built. This is for example the case of the espresso algorithm implemented in LigandScout [26,27], which achieved the pharmacophore modelling by dividing the procedure previously described into six steps exploiting various algorithms and two alignment methods: the first, based on a combination of shared pharmacophore feature and the second, based on a merged pharmacophore feature. Finally, the common features present in all training compounds are included in the common features models and all features of the aligned compounds are summated in a merged feature pharmacophore [28]. Another example of similar functionality is given by the HipHop algorithm provided by DiscoveryStudio [29], which builds several pharmacophore models starting from identifying the common chemical features arrangement of the training set structures. Beginning with a few groups of features, which are extended until no more shared configuration features are found, several models are obtained and ranked based on the fitness of the training compounds. Models are refined by the HipHop Refinement algorithm, which adds space restrains imposed by the structures of inactive compounds.

QSAR (Quantitative structure-activity relationships) based pharmacophore is a mathematical model that tries to find statistical correlations between structures and functions, quantifying the impacts in a biological activity of specific structural modifications in existing or predicted molecules [30]. Based on the idea that molecules with similar physico-chemical properties show a similar binding affinity for a protein target, nowadays the QSAR approach is used in drug discovery to build statistical models derived from these correlations exploiting for the prediction of the bioavailability, the ADMET profile (toxicity), adsorption, distribution, metabolism, binding affinity, biological activity and elimination of novel compounds [31].

**Figure 3:**
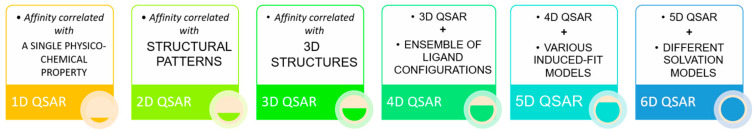
QSAR classifications. The QSAR approaches are classified from the 1D to the 6D-QSAR according to the different dimensions of the descriptors implemented in the method.

There are six classifications of the QSAR approaches, from the 1D to the 6D-QSAR, according to the different dimensions of the method (Figure 3).

1D-QSAR takes into account a single physico-chemical property of the ligand, for an example the pKa value. In the 2D-QSAR, instead, affinity is correlated with structural patterns, while in the 3D-QSAR with the 3D structure of the ligand and its interactions. The concept extends as dimension rises: 4D-QSAR incorporates an ensemble of ligand configurations in 3D-QSAR, 5D-QSAR adds to 4D-QSAR various induced-fit models, 6D-QSAR implements 5D-QSAR with different solvation models [25].

### Pharmacophore Models Validation

Once one or more pharmacophore models have been computed, a validation step is crucial before their implementation for practical purposes. Pharmacophore validation could be performed by exploiting several methods, such as the goodness of hit list (GH), receiver operating characteristic (ROC) curves construction, Fischer’s method, or other statistical analysis, which relies on screening a test set and decoy set (if needed) to evaluate the model ability to distinguish active and inactive molecules and provide an estimation of its quality [9]. 

Mainly, the quality of a model can be described by four parameters: the sensitivity (capacity to detect active compounds), the specificity (capacity to exclude the inactive molecules), the yield of actives (the ratio between true positives and the number of hits) and the enrichment factor (which relates the yield of actives to the composition of the screening dataset) [7].

The GH scoring method consists of calculating the percentage of sensitivity, the percentage of the yield of actives the enrichment factor and the Güner–Henry score, which gives an evaluation of the efficiency of the screening dataset search and can vary from 0 to 1 where 1 is the value for the ideal model [32]. The ROC curve shows the enrichment power of a model plotting the sensitivity against 1—specificity (the false positive rate). The area under this curve (AUC) gives a measure of the pharmacophore’s performance and it is useful for multiple models evaluation. AUC can vary from 0 to 1, where 1 corresponds to an ideal case in which all the active compounds are detected at first, 0 to the collection of the inactive ones at first and 0.5 to random results [33]. Fisher’s randomization test validation method is instead used to analyze the significance of the statistical correlation between structure and biological activity [34]. Regression analysis can also be applied to check for the correlation between the compounds predicted as active and those whose bioactivity is experimentally confirmed.

In case the validation process reveals low-quality results, manual refinement could be a way to improve its performance by applying some changes in chemical features’ selection or definition settings, training or test sets composition or in the pharmacophore building procedure, but a new model generation with a different approach and algorithm should not be excluded.

## 4. Pharmacophore-Based Virtual Screening

Among the strategies for the identification of new bioactive substances, VS techniques play a prominent role. VS tools are important in drug discovery as they increase the speed of the bioactive molecule discovery process through computational simulations by selecting from large libraries the compounds that are most likely to interact with the identified target. In addition, VS identifies compounds that may be toxic or have unfavorable pharmacodynamic (for example, potency, affinity, selectivity) and pharmacokinetic (for example, absorption, metabolism, bioavailability) properties [35]. 

In this context, pharmacophore models can be successfully applied to filter large collections of compounds to find the so-called hits, i.e., novel molecules matching the pharmacophoric features required to be potentially active against a specific target. Since a pharmacophore does not represent exact chemical groups but chemical functionalities and their spatial relationships, the retrieved hits usually include structurally different compounds, making pharmacophores useful tools for scaffold hopping [7]. 

The pharmacophore-based screening can be performed directly using some of the software already mentioned for the pharmacophore generation, which allows doing this on a manually created dataset. Other useful open-access platforms for virtual screening are Pharmit (http://pharmit.csb.pitt.edu) (accessed on 20 May 2022) and ZINCPharmer (http://zincpharmer.csb.pitt.edu/) (accessed on 20 May 2022). The first one allows the user to import a predefined pharmacophore query or elucidate pharmacophore and shape queries from the receptor and/or ligand structures, and to use it to screen a desired dataset among a set of provided compounds libraries or a personal one. The results are quickly computed and classified according to different criteria such as energy minimization [36]. ZINCPharmer is a user-friendly online web server, which, exploiting the Pharmer research technology, allows screening of the purchasable molecules present only in the ZINC database using a pharmacophore model imported from other tools or directly derived from ligands or structures [37].

**Figure 4:**
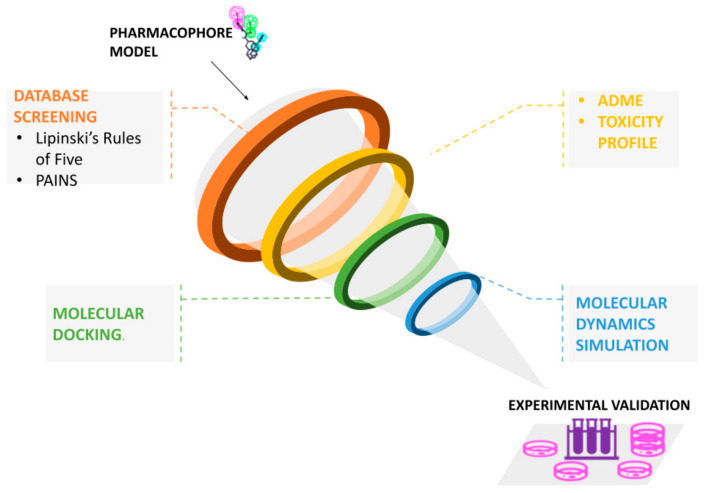
Virtual screening workflow. Basic filters and methods applicable for a virtual screening process. Scheme of the multistage vs. approach. The application of vs. follows a typical sequence of processes with a cascade of filters able to narrow down and choose a set of hits with potential biological activities.

In general, once the model to use has been defined, the first step in virtual screening is to consult a database that contains a large number of compounds annotated with information about the 3D structure, known target, and in some cases the purchasability. Some of the free databases include the Protein Data Bank (PDB) [38], PubChem [39], ChEMBL [21], Zinc [40], and Drugbank [22]. Moreover, there are some commercially available databases such as the MDL Drug Data Report. Usually, due to the presence of a multitude of compounds in these repositories, the strategy adopted by many researchers is to filter the data by applying different parameters to reduce the computational cost of the pharmacophore searching. This may be obtained through the exclusion of compounds that are too big for the ligand pocket, the use of Lipinski’s Rule of Five or standard metrics for lead-likeness, and removing compounds deemed to be pan-assay interference compounds (PAINS) [35,41]. In addition, a combination of filtering for desired pharmacological and adsorption, distribution, metabolism, excretion, and toxicological (ADMET) properties is advisable to be applied early in the virtual screening process [42]. These filters should be considered suggestions, not mandatory, and they can be applied depending on the specific case. As an example, the Rule of Five is not an absolute rule, as many drugs go under the “beyond the rule of five” [43]. To efficiently filter a library of compounds against such criteria, several online tools have been developed. For example, the commercial software ACD/LogD Suite (https://www.acdlabs.com/products/percepta-platform/physchem-suite/logd/) (accessed on 20 May 2022) by Advanced Chemistry Development (ACD/Labs) can be used to predict ADME-related properties including hydrophobicity, lipophilicity and pKa, while Pharma Algorithms provided a suite of products via ADME Boxes (the company joined ACD/Labs and the ADME Suite is now available at https://www.acdlabs.com/products/percepta-platform/adme-suite/) (accessed on 20 May 2022) that addresses issues such as solubility, oral bioavailability, absorption and distribution [1]. It is necessary to underline that in some protocols reported in the literature, the evaluation of toxicity and of the ADME profile is made only after molecular docking. Therefore, it is in the choice of the researcher the use of a protocol that adds a preliminary filter, thus reducing the number of molecules on which to apply further analysis or proceed on a larger number of molecules to identify potential ligands that, if not usable directly because of their toxicity, could serve as the basis for the synthesis of new molecules. If the structure information of the target is available, the retrieved pool of compounds can be further selected by molecular docking with programs such as DOCK [44] or Autodock (http://autodock.scripps.edu/) (accessed on 20 May 2022) [45], to have a preliminary evaluation of binding affinities and exclude molecules with very low values, as well as to obtain preliminary protein-ligand complexes. Molecular docking is a computational process where ligands are moved in 3D space to find a configuration of the target and ligand that maximizes the scoring function [1]. With docking, therefore, ligand–target complexes are obtained and can be evaluated more in depth, by specific methods, to verify if these compounds can be used subsequently for in vitro experimentation. One of these evaluation methods is the molecular dynamics (MD) approach. It is the pivotal theoretical approach, which can be utilized to gain molecular insight into the stability of the binding pose of the screened molecules in the active site [46], giving a more accurate evaluation of their binding affinity. At the end of the virtual screening, the selected compounds matching the desired properties and giving the best results must undergo an in vitro validation process to verify if indeed the results obtained in silico are reliable. Everything said up to now represents a typical workflow followed in a virtual screening analysis and it is graphically depicted in Figure 4.

Recently, MD simulations have been used to perform a thorough conformational search without any help of traditional docking procedures, with accurate all-atom force field and enhanced sampling techniques, obtaining accurate results for both binding modes and binding affinities [47,48].

Finally, it is necessary to also mention the free energy calculations used to obtain protein–ligand binding affinity by MD simulations in conjunction with molecular docking [49,50,51,52].

## 5. Limitations and Possible Solutions

As shown, pharmacophore modelling is a very useful tool for many applications in drug discovery or drug design. However, despite its strength, this method is not free of limitations that have implications in virtual screening increasing the rate of false positives and/or false negatives, and that should be in mind to find proper solutions, when possible, and to have a critical view of the results.

As explained in the previous paragraphs the quality of a pharmacophore model is strictly related to that of the input dataset and the algorithm used for the modelling. Indeed, the quality of the structures used for pharmacophore building, refinement, and validation greatly affect its reliability, and to overcome this limit many authors suggest a manual inspection of the structures used and of the annotations regarding the biological data associated with them. For what concerns the choice of the algorithm, it is important to underline that different algorithms can give different results with no overlap starting from the same macromolecular target. This could be attributed to the different screening methodology of the algorithm, but also to the specific screening dataset used among the several publicly available screening platforms [7]. Therefore, an integration of different tools is recommended to cover a larger chemical space and obtain different chemotypes.

Moreover, whether the structure-based or the ligand-based approach is used, both present some intrinsic limitations. In particular, flexibility is the main drawback of the structure-based strategy. As already explained, a structure-based pharmacophore derives from the 3D structure of a macromolecule target, ideally complexed with an active compound. A single structure represents a static image reproducing a single binding mode of a ligand towards its target without taking into account the dynamic nature of the system due to receptor and ligand conformational flexibility [53]. This lacking information results in pharmacophore models that may be defective in some features that could be relevant for the binding of different ligands and, therefore, in retrieving new potential hits. If experimental structures of the same protein target complexed with different ligands exist, it is possible to reduce this limit by using them to extract and merge features responsible for ligands interactions into a more refined model. However, this does not often happen and an alternative way to handle the flexibility consists in employing computational techniques such as flexible docking and/or MD simulations of the investigated system. Dynamic pharmacophores can be computed from protein-ligand MD trajectories and have been successfully applied in recent studies showing a better performance in the virtual screening of bioactive compounds compared to the classic rigid approach [54,55,56]. Nevertheless, this methodology needs higher computational resources (a problem that is less relevant due to the increasing availability of computing resources, and in case of application to a small set of selected ligands) and expert knowledge since it produces many models that should be averaged or clustered to derive more representative pharmacophores [57].

Another common problem affecting the structure-based approach is the detection of a high number of unprioritized pharmacophoric features, especially when apo-structures are the only available starting point. In this case, the derived pharmacophore model is too restrictive for virtual screening making the identification of structurally different compounds with similar characteristics difficult [17]. Given this, the reduction of chemical features is crucial to obtaining a reasonable and usable model. Some strategies to apply for this purpose have already been discussed, but they depend on available data and an expert manual intervention is often required.

Regarding the ligand-based method, the major limitation is the absence of receptor complementary information related to the interaction pattern with the binding pocket or even just about its shape. By its nature, the ligand-based approach is founded on all possible chemical features and geometric information inferable from the input compounds and their flexible alignment. However, more are the chemical features to be taken into account by the algorithm, more is the time cost of it. Therefore, this approach is limited to small compounds and simple chemical features by computational/time costs, generating a less tailored pharmacophore model [27].

A further complication for the ligand-based modelling is the low availability of inactive compounds, due to the lack in the literature of negative results. The use of a not enough number of molecules known as inactive for a specific protein target has an impact on the selectivity and sensitivity of the pharmacophore model obtained [6].

Above the individual deficiencies, another key point to place is that the pharmacophore represents a model of the chemical features but other variables that can influence the binding of active compounds to a specific target, such as chemical solubility, cell metabolism, membrane permeability, the particular toxicologic effect could not be considered or well-integrated on it; this could have a strong impact on the real capacity of the selected compounds to have a suitable biological effect on the target [7].

In general, even if some of these issues could be addressed by the use of accurate structures, training and test sets, the validity of the model obtained should be always compared to the statistical robustness of the input dataset, because no matter how complete the dataset employed could be, no model can be judged as universal [58].

Because of the things reviewed above, the simultaneous use of both strategies, when it is allowed, is advantageous since the weaknesses of one can be balanced by the strengths of the other. Moreover, their combination with multiple approaches at different stages, such as MD simulation, docking studies and machine learning approaches, could be a valuable resource to overcome the inherent limitations of the pharmacophore modelling, improving the chance to have more complete and reliable results.

## 6. Software for Pharmacophore Modelling

Several articles reported lists of software, databases and online tools for CADD applications [1,25,59]. Moreover, online services and catalogues provide links to many resources (see https://www.click2drug.org/ or https://bio.tools/) (accessed on 20 May 2022). In this paragraph, we report the most used and reliable, in our opinion, and findable at the time of the writing of this review. Several programs have been developed to perform the pharmacophore modeling in an automated way using different algorithms, such as LigandScout, Schrodinger-Maestro, MOE, and Discovery Studio. Among them, LigandScout [60] is widely used for pharmacophores generation from a protein–ligand complex and performs a step-by-step ligand topology interpretation including aromatic ring detection, the assignment of functional group patterns, the determination of hybridization state and bond types, followed by ligand and binding site analysis to automatically detect and classify protein–ligand interactions into hydrogen bonding, ionic, aromatic and lipophilic contacts according to geometric constraints. The ligand features composing this set of paired interactions define a pharmacophore model which can be also modified by removing specific features [60]. The Schrodinger–Maestro suite allows the generation of e-pharmacophores, energetically-optimized pharmacophores using the Glide XP scoring function to prioritize protein-ligands interaction sites and shows good speed and performance in virtual screening [61]. MOE is an integrated computer-aided molecular design platform that offers many different services, from drug design to virtual screening and molecular simulations. Drug design by MOE allows exploiting different methods such as the structure-based design, the ligand-based design and the fragment-based discovery (www.chemcomp.com/MOE-Molecular_Operating_Environment.htm/) (accessed on 20 May 2022). Another commonly used software is Discovery Studio (BIOVIA, Dassault Systèmes), which allows deriving structure-based and/or ligand-based pharmacophores. In the first case, it offers three methods to build pharmacophores: structure-based, fragment-based or receptor-ligand pharmacophores according to the available structural data. In absence of a bound ligand, this program implements the Interaction Generation protocol to create LUDI interaction maps for hydrogen and hydrophobic contacts with the binding site that can be manually selected or computationally computed. The interaction map is then converted into pharmacophore models consisting of different combinations of at least three features complementary to those of residue in the binding pocket in terms of chemical nature and location, and the resultant models are scored and ranked according to their target selectivity [62]. For ligand-based pharmacophore generation, besides the use of the HipHop algorithm, DiscoveryStudio gives also the opportunity to explore the QSAR approach by the HypoGen algorithm [63]. The purpose of this algorithm is to find a spatial 3D arrangement of the features shared by the training molecules whose activity on a specific target has been measured. To do so, it needs as input at least 16 compounds covering four orders of magnitudes: H-bond donors, H-bond acceptor, aromatic ring, hydrophobicity. The algorithm works in three phases: the constructive, the subtractive and the optimization. In the first phase, all the pharmacophoric conformations, which preserve at most five default features, are collected and pharmacophores preserving features shared by all the bioactive training compounds are generated; only pharmacophores fitting other active molecules are kept. In the second phase, pharmacophores fitting inactive molecules are removed; in the last, pharmacophore collection is optimized by simulating annealing algorithm. Only the pharmacophore models showing the highest score are given as output [7]. Other examples of 3D QSAR programs, which generate predictive models include PHASE [64], comparative molecular field analysis (CoMFA) [65] and comparative similarity indices analysis (CoMSIA) [66]. For a more exhaustive list, we remand to Gurung et colleagues’ review of 2021 [26]. 

The software tools mentioned above are commercial but there are also academic programs freely accessible. For instance, Pocket v.2 is a structure-based pharmacophore program available either on the web (http://www.pkumdl.cn:8080/pocketv2.html) (accessed on 20 May 2022) or a stand-alone version that uses the Pocket module of LigBuilder (for de novo structure-based ligand design) to analyze the binding site of the target receptor by scored grids. The basic required input is the protein 3D structure in PDB format that is employed to generate pharmacophore models from receptor structures with or without a bound compound [67]. 

A user-friendly and freely-available web server, which exploited the common feature approach for ligand-based modelling, is represented by PharmaGist [68]. The main window of PharmaGist requires only the uploading of up to 32 input molecules in mol2 format (uploadable in single or in a unique zip file), with bonds angles and length already corrected and hydrogens atoms explicitly specified, the selection of the number of output of pharmacophores that will be showed, and an e-mail address where the link to the results, stored at least for a month, will be sent. Besides this standard research PharmaGist gives also the possibility to choose some advanced options as: i. the selection of a key molecule, by this option all the other molecules will be aligned to the compound selected, otherwise all compounds are selected as key compounds; ii. the minimum number of features in pharmacophore iii. the feature weighting options, by which it is possible to modify the weight of some features (aromatic ring, hydrogen bond donor/acceptor, anion/cation and hydrophobic properties) in the scoring function; iv. User-defined features, by which additional feature types can be defined by a supported feature file format created by the user. The output consists of several tables: the first gives a summary of the input molecules’ features and the following summarized the results starting always with the highest scoring pharmacophore.

For those who are researching an easy to use and free available tool which exploits the QSAR method without requiring particular informatics skills, or who simply want to start learning about pharmacophore construction, the web portal 3d-qsar.com (accessed on 20 May 2022) represents a valid opportunity, being a platform hosting several applications which guide the user along the correct workflow necessary to build a pharmacophore model. The first is represented by Py-MolEdit which allows the correct compilation and uploading of the training and the test dataset in any format, giving also the possibility to draw your molecules. After using that tool, the platform gives the discretionary option to align your dataset by exploiting the Py-Align tool. At this point is possible to perform the building of a pharmacophore model with Py-CoMFA, obtained which, can be easily analyzed by Py-ConfSearch tool that performs a conformational analysis. Py-CoMFA allows the building and validation of the 3-D QSAR model with the same style as the original CoMFA software, which seems to reproduce published results with a very good agreement. The platform also provides the opportunity to make docking simulations (by Py-Docking), or to use a different QSAR algorithm (Py-ComBinE). An interface to the Protein Data Bank is also provided (by Py-PDB) [69].

## 7. Examples and Case Studies

Many examples in the literature present pharmacophore models obtained by QSAR and common feature pharmacophore approaches. In addition to articles and reviews published in the past that report on successful applications in pharmacophore modeling and virtual screening [70,71,72,73,74], we highlight some specific example of studies.

Schuster and colleagues performed the ligand-based pharmacophore modelling for selective and nonselective 11β-HSD1 inhibitors. Their pharmacophore models, after a virtual screening procedure, led to the in vitro validation of 30 compounds among which seven were found to inhibit more than 70% of the activity of 11β -HSD1 when measured in cell lysates with IC50 values below 10 µM and without cytotoxicity at concentrations up to 40 µM [75]. Pal and colleagues exploited the 3D-QSAR pharmacophore hypothesis and found relevant binding features of novel topoisomerase inhibitors [46].

In a recent study, a pharmacophore model based on the experimental structure of AKT1 protein complexed with an inhibitor has been used for virtual screening on a database of natural compounds. The most promising compounds obtained by the screening have been further investigated by toxicity profile and ADMET analyses, together with molecular docking for better prediction of the potential binding [76]. Experimental studies confirmed the potential inhibitory role of at least one of the selected compounds [76] and, in addition, its synergic effect with quercetin to inhibition of cell growth and induce apoptosis [77].

Due to the pandemic, many research teams explored the opportunity to find novel drugs through time-saving CADD approaches. This is the case of a study oriented to finding novel inhibitors of TMPRSS2, or type II transmembrane serine protease, a human protein involved in the activating processes of SARS-CoV-2 [78]. TMPRSS2 is of particular interest for its involvement also in cancer pathologies. The in silico screening has been performed by a pharmacophore-based approach, starting from camostat mesylate, a known inhibitor of serine protease 2, also approved for drug. The authors selected 10 pharmacophoric features of camostat mesylate and selected 2140 compounds from a public database containing more than 30,000 natural compounds. After a molecular docking analysis, the 2140 compounds were filtered to 85 candidates with docking scores similar to or better than the known inhibitor. The computational study has been further extended to analyze the list of filtered compounds for matching with Lipinski’s rule of five and for ADMET properties. The study has been cited many times in literature, due to the interest in the results and the need to verify by experimental approaches the real potential inhibitory activity of filtered compounds.

## 8. Future Perspectives

CADD approaches have been successfully developed to improve the ability to discover new drugs. The increasing computational power of hardware opens the perspective of many more applications, also by increasing the availability of protein target structures through deep learning and AI-based prediction tools [79]. The deep learning approaches have been also recently applied to drug-target interaction [80]. However, the experimental steps needed to validate the compound activity and safety remain time-consuming and will always slow down the whole drug discovery process. From this point of view, the pharmacophore modelling followed by the virtual screening of databases of approved drugs, in the drug repurposing perspective, represents the most advanced rational approach, and the most promising way to find novel therapies.

## Data Availability

No new data were created or analyzed in this study. Data sharing is not applicable to this article.
